# Supplementary material for: Targeting IL-21 to tumor-reactive T cells enhances memory T cell responses and anti-PD-1 antibody therapy
Source: Nat Commun. 2021 Feb 11;12:951. doi: 10.1038/s41467-021-21241-0 (PMC7878483; doi:10.1038/s41467-021-21241-0)
Supplement: Supplementary file 1 — Supplementary Information [file 41467_2021_21241_MOESM1_ESM.pdf]

Supplementary Information

Targeting IL-21 to tumor-reactive T cells enhances memory T cell responses and anti-PD-1 antibody therapy

The PDF file includes:

Fig. S1. Differentiation of activated $CD8^+$ T cells induced by different cytokines or proteins

Fig. S2. Characterization of naïve-like $CD8^+$ T cells induced by PD-1Ab21

Fig. S3. Differentiation of T_{SCM}-like cells induced by different forms of IL-21

Fig. S4. Potent antitumor effects of PD-1Ab21 in various tumor models

Fig. S5. Anti-tumor effects of PD-1Ab21 in various tumor models treated by hydrodynamic injections

Fig. S6. Analysis of the targeting of PD-1Ab21 to tumor-specific T cells in vivo.

Fig. S7. Flow cytometry analysis of immune cells in the treated tumor-bearing mice

Figure S8. Flow cytometry analysis of tumor-specific T cells in the treated tumor-bearing mice.

Supplementary Figures

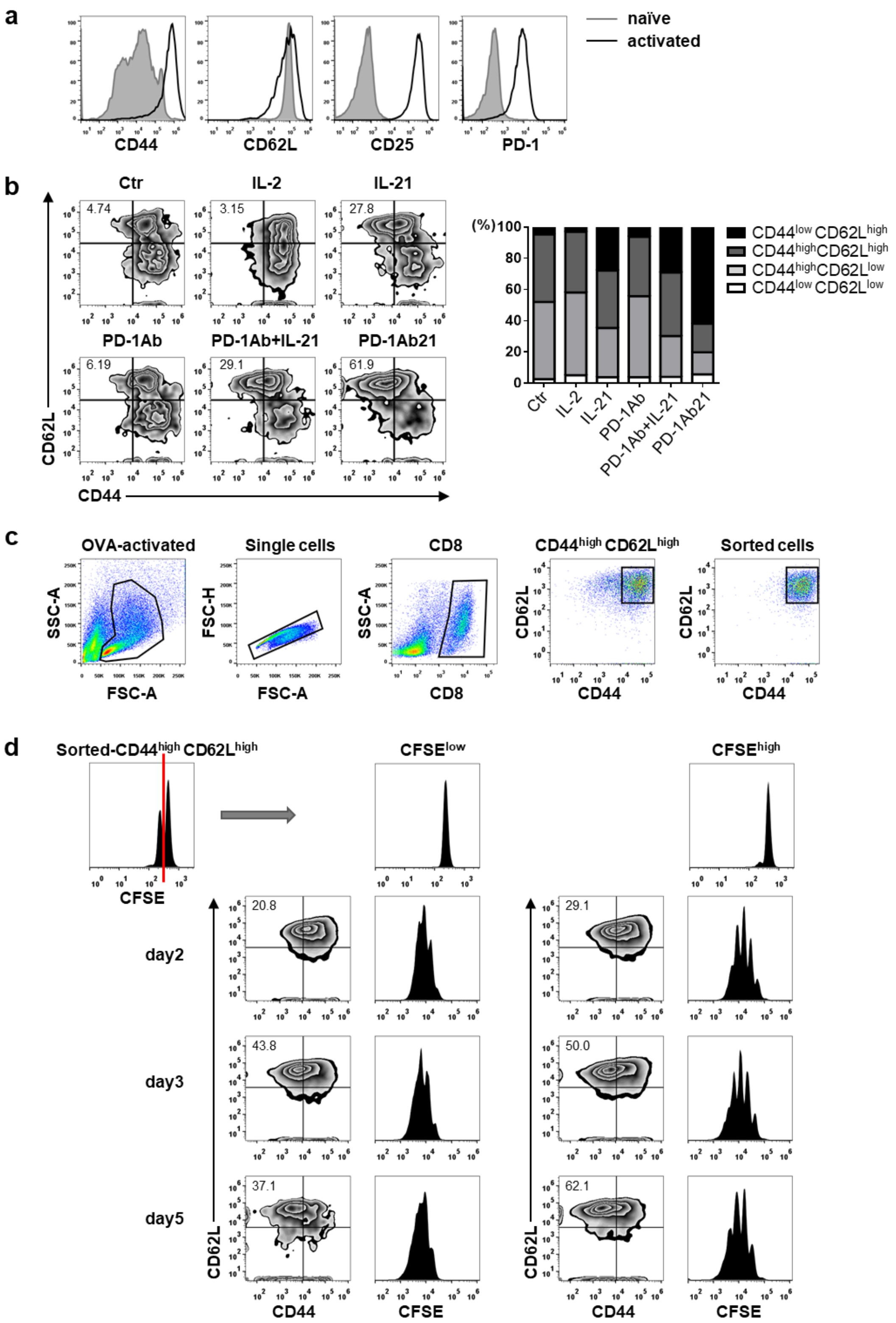

e

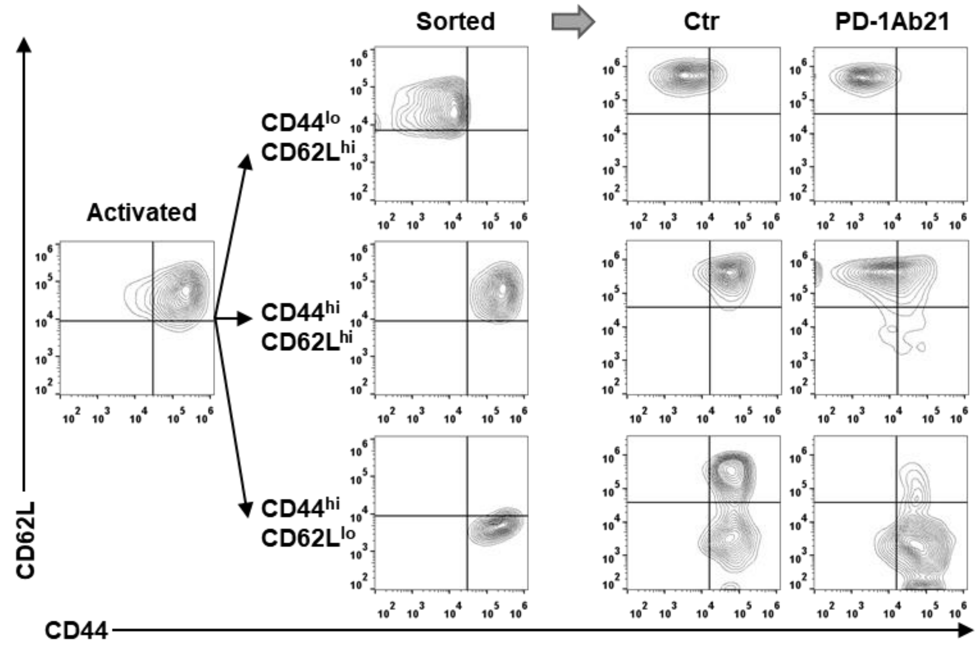

Supplementary Figure 1. Differentiation of activated CD8$^+$ T cells induced by different cytokines or proteins. (a) Flow cytometry analysis of surface markers of naïve (gray histogram) and OVA$_{257\text{-}264}$-activated OT-1 cells (white histogram). **(b)** Flow cytometry analysis of the differentiation of T cells after activation with anti-CD3 and anti-CD28. Naïve T cells from LNs were activated with anti-CD3 and anti-CD28 for 40 hours, then differentiated with the indicated cytokines or proteins for 3 days. T cell differentiation was analyzed by expression of CD62L and CD44 on gated live CD8$^+$ T cells (left). Column graphs show the relative frequencies of different cell populations (right). **(c)** Sorting strategy for CFSE-labeled OVA-activated CD44^{high}CD62L^{high} OT-1 cells. All data are representative of at least three independently performed experiments. **(d)** Relationship between proliferation and differentiation of activated CD8$^+$ T cells. OVA-activated CD44^{high}CD62L^{high} OT-1 cells were sorted into CFSE^{high} and CFSE^{low} populations. Proliferation and phenotypes of T cells were monitored by flow cytometry during T cell differentiation. **(e)** Differentiation of different activated CD8$^+$ T cell populations. Naïve OT-1 cells were primed with OVA$_{257\text{-}264}$ for 2 days. Then, the different cell populations were sorted and differentiated with or without PD-1Ab21 for 3 days. T cell differentiation was analyzed by the expression of CD62L and CD44 on gated live CD8$^+$ T cells. Numbers show the percentage of cell population in the dot-plot quadrants. All data are representative of at least three independently performed experiments.

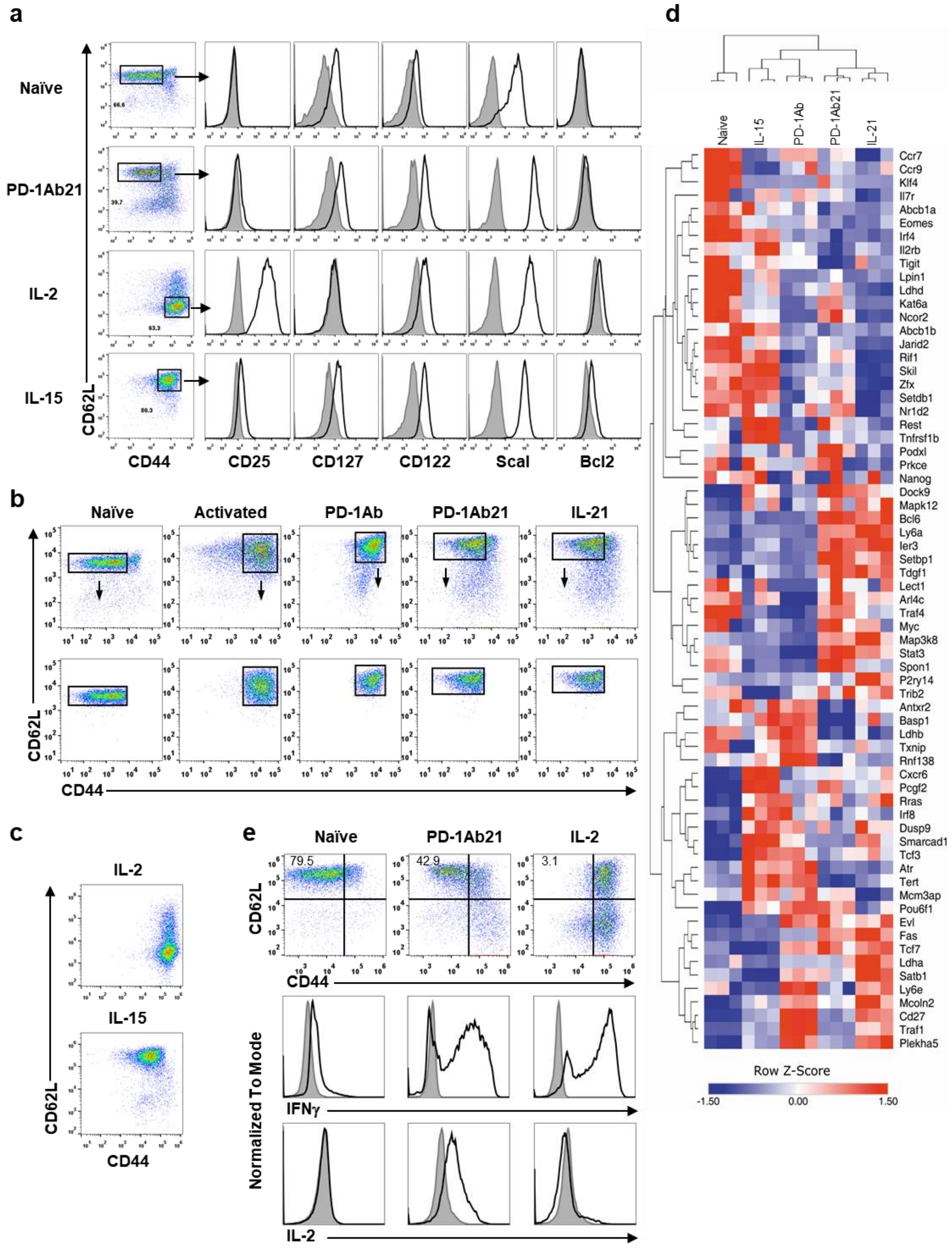

Supplementary Figure 2. Characterization of naïve-like CD8$^+$ T cells induced by PD-1Ab21. (a) Flow cytometry analysis of the expression of surface markers on naïve T cells, PD-1Ab21-differentiated naïve-like T cells, IL-2-differentiated T_E/T_{EM} and IL-15-differentiated T_{CM}. CD8$^+$ T cells were gated and the different cell

populations were identified by the expression of CD44 and CD62L. Results with the isotype antibody (gray histogram) and antibodies against different phenotypic markers (white histogram) are shown. Data are representative of at least three independently performed experiments. **(b,c)** Phenotypes of T cells prepared for RNA-seq. Gating strategy for sorting of T_N, activated T cells and PD-1Ab-, IL-21-, PD-1Ab21-differentiated T cells (b). Phenotypic analysis of IL-2- or IL-15-induced OT-1 cells(c). **(d)** Heatmap showing normalized expression levels of the genes with a $CD8^+$ T stem cell-like memory signature described by Luigia Pace et al. 2018. **(e)** Recall responses of the differentiated T cells. IL-2- and PD-1Ab21-differentiated OT-1 cells were rested in IL-7 for 2 days, and then stimulated with anti-CD3 antibody overnight. BFA was added to the medium 4 hours before IFN-γ and IL-2 intracellular staining was performed. Naïve OT-1 cells were used as control. Phenotypes of naïve, IL-2-differentiated and PD-1Ab21-differentiated OT-1 cells are shown (top). The data are representative of at least three independently performed experiments.

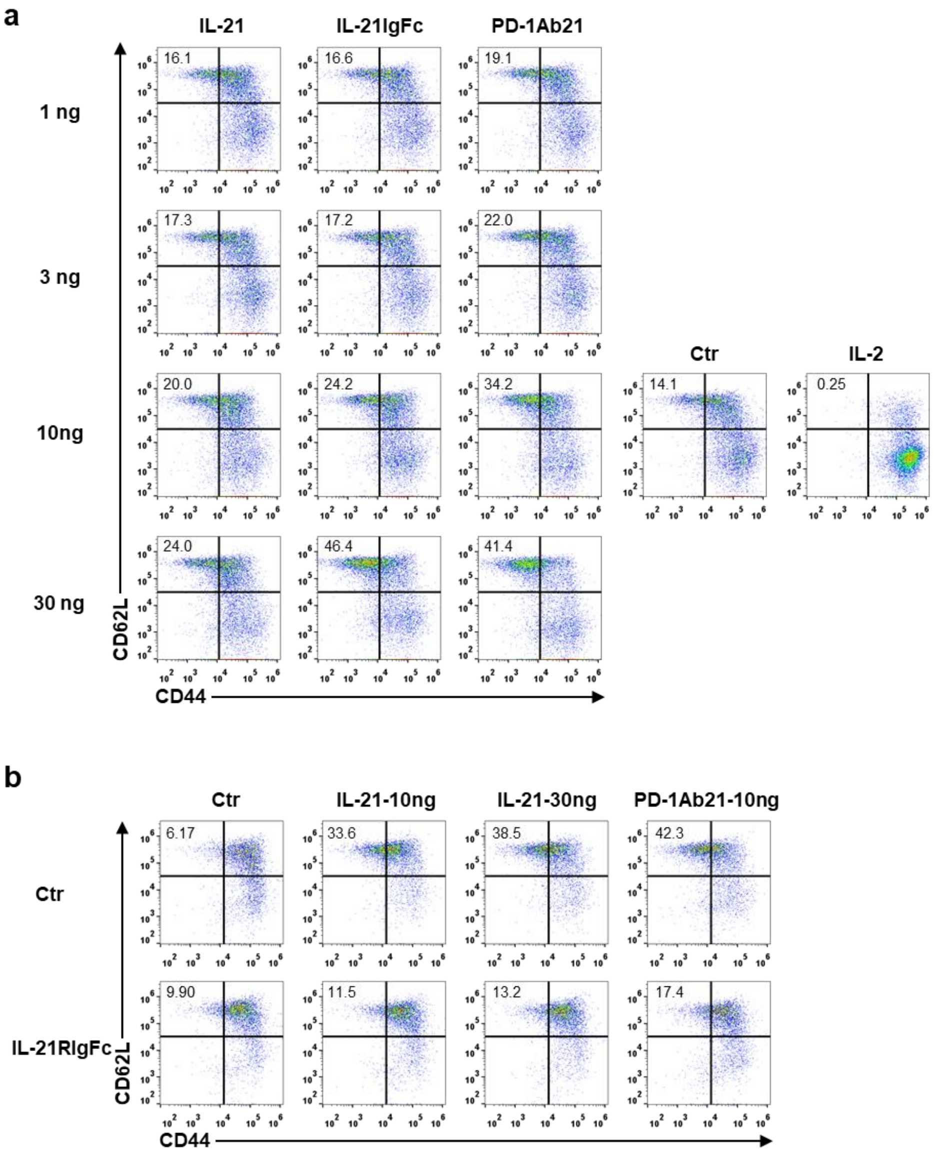

Supplementary Figure 3. Differentiation of T_{SCM}-like cells induced by different forms of IL-21. Naïve OT-1 cells were activated by OVA$_{257\text{-}264}$ peptide for 2 days and then differentiated with the indicated concentrations of cytokine or fusion proteins **(a)**, or with mixtures of IL-21RIgFc and IL-21 or PD-1Ab21, which had been pre-incubated at 4°C for 2 hours before addition to the cell culture **(b)**. The phenotypes of T cells were analyzed by flow cytometry 3 days after differentiation. Representative dot-plots of phenotypes of differentiated T cells are shown. Numbers show the percentage of CD44^{low}CD62L^{high} T cells in the dot-plot quadrants. All data are representative of at least three independently performed experiments.

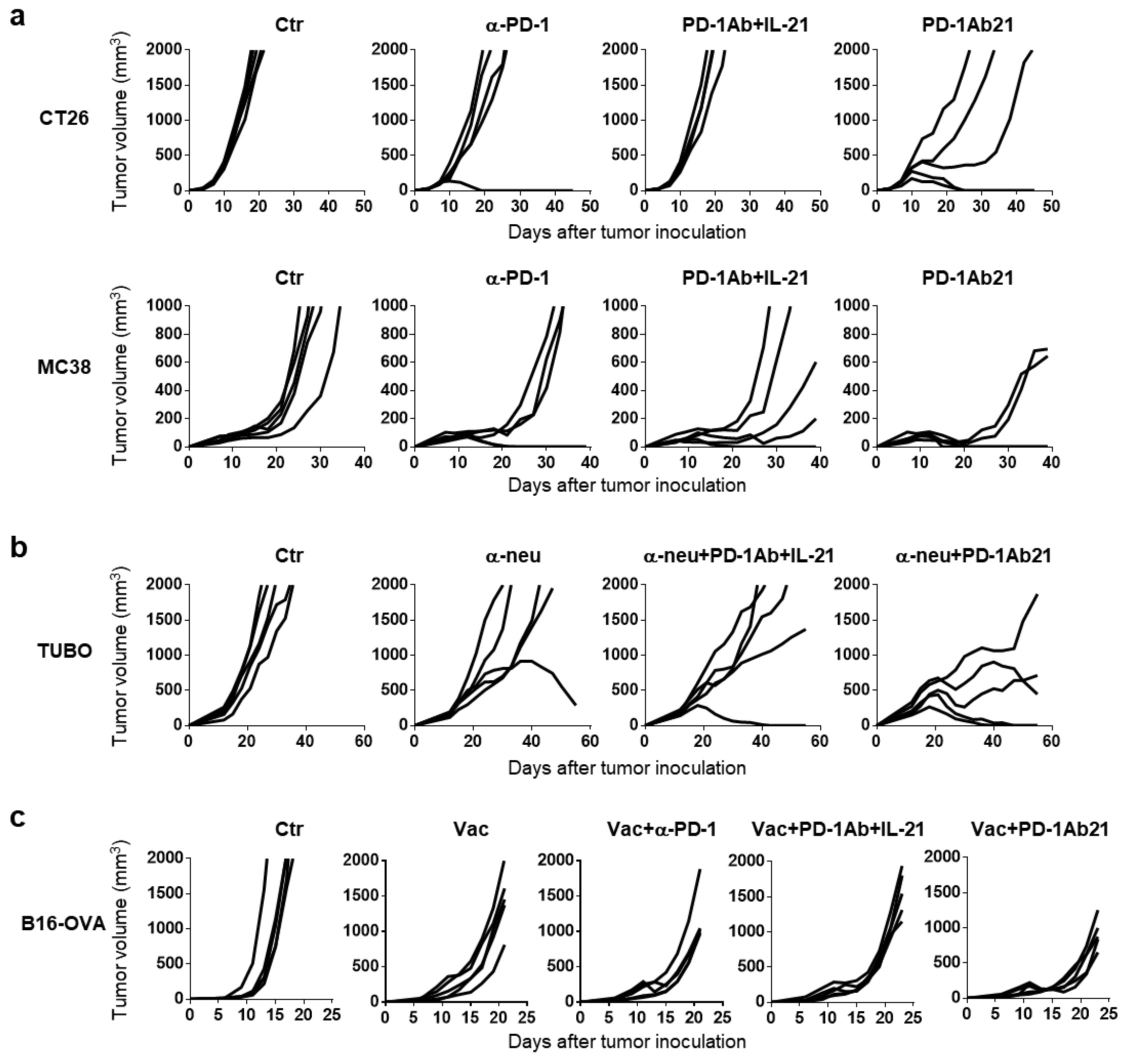

Supplementary Figure 4. Potent antitumor effects of PD-1Ab21 in various tumor models. **(a)** Balb/c mice transplanted s.c. with CT26 cells (upper row) or C57BL/6 mice transplanted s.c. with MC38 cells (down row) were treated with anti-PD-1 antibody, PD-1Ab + IL-21, or PD-1Ab21 (n=5 mice /group). **(b)** Balb/c mice (n=5 mice /group) transplanted s.c. with TUBO cells were treated with anti-Her2/neu antibody alone or in combination with PD-1Ab + IL-21 or PD-1Ab21. **(c)** C57BL/6 mice (n=5 mice /group) transferred with 1×10^6 naïve $CD90.1^+$ OT-1 cells 1 day before inoculation of B16-OVA cells were immunized with poly I:C and $OVA_{257\text{-}264}$ peptide, followed by treatment with anti-PD-1, PD-1Ab + IL-21, or PD-1Ab21. Tumor length (a) and width (b) were measured and tumor volume was calculated as ($ab^2/2$). One representative experiment out of at least three independent experiments is shown.

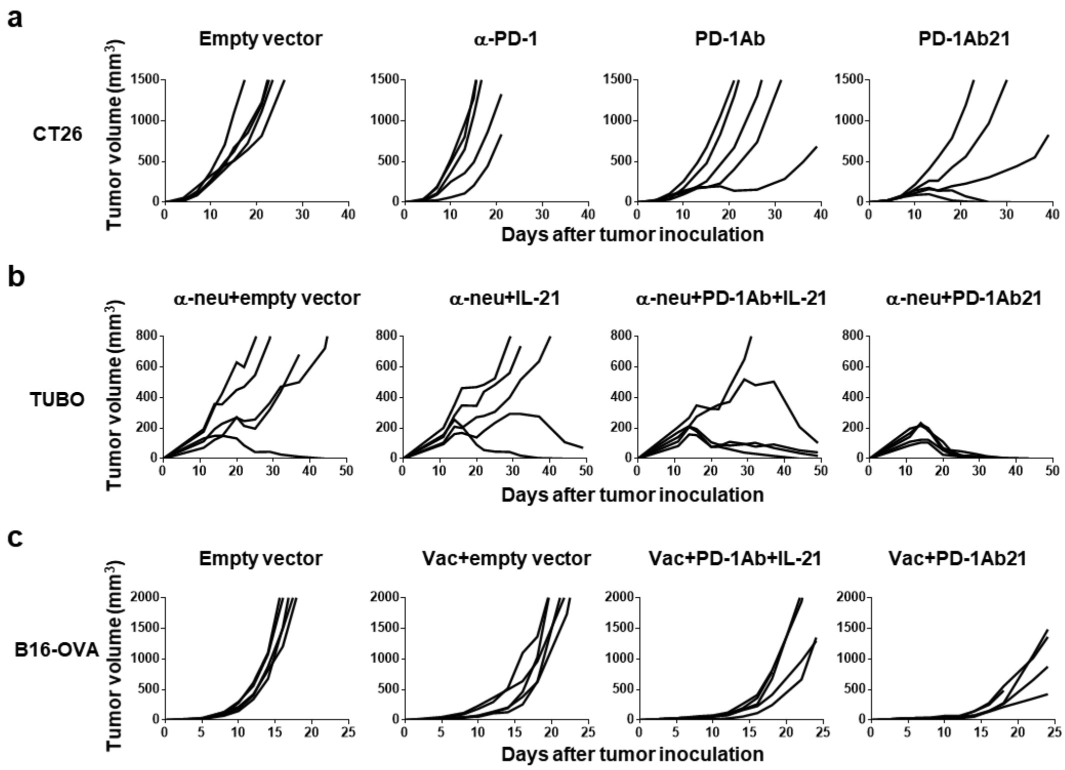

Supplementary Figure 5. Antitumor effects of PD-1Ab21 in various tumor models treated by hydrodynamic injection. (a) Balb/c mice (n=5 mice /group) transplanted s.c. with CT26 cells were treated with i.p. injection of anti-PD-1 antibody, or hydrodynamic injections of the indicated plasmid on day 4 and day 9 after tumor inoculation. **(b)** Balb/c mice (n=5 mice /goup) transplanted s.c. with TUBO cells were treated i.p. with anti-neu on day 13 and day 16 after tumor inoculation. Hydrodynamic injections of the indicated plasmids were performed on day 12. **(c)** C57BL/6 mice (n=5 mice /group) were transferred with 1×10^6 naïve OT-1 cells 1 d before inoculation (s.c.) of B16-OVA cells. 3 days after tumor inoculation, mice were immunized with $OVA_{257-264}$ peptide mixed with Freund's complete adjuvant. Hydrodynamic injections with the indicated plasmids were performed on day 4 and day 10. Tumor volumes were calculated as ($ab^2/2$), where (a) is the length and (b) is the width. One representative experiment out of at least two independent experiments is shown.

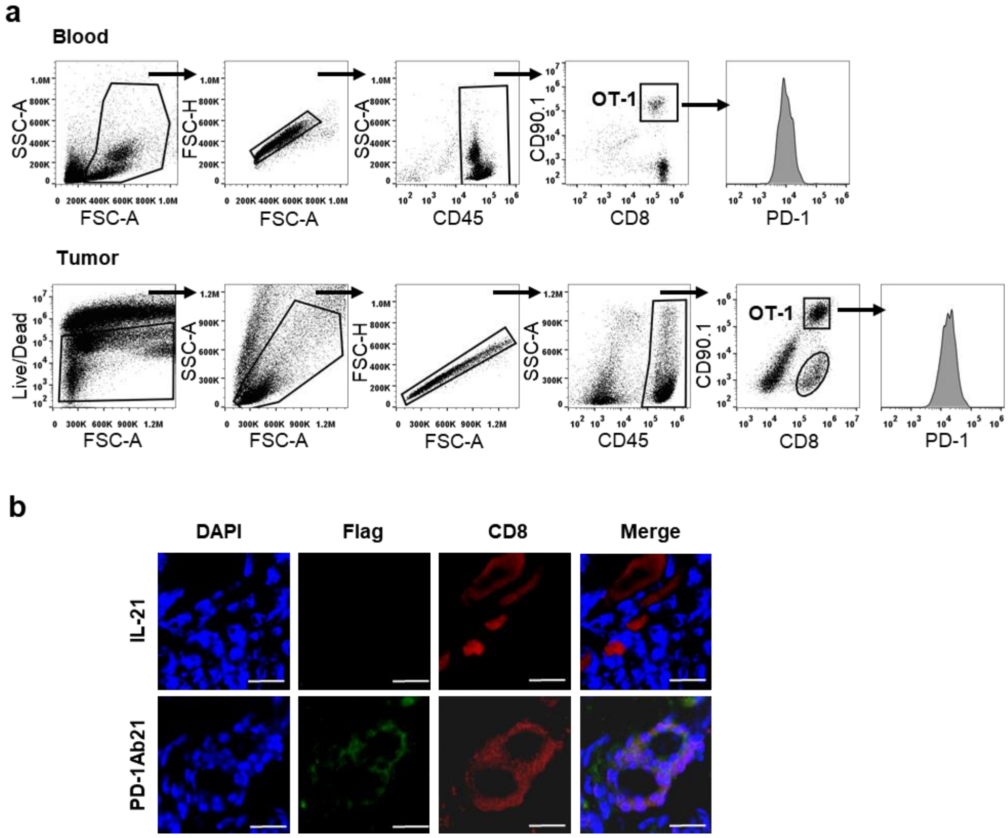

Supplementary Figure 6. Analysis of the targeting of PD-1Ab21 to tumor-specific T cells in vivo. (a) Gating strategy of PD-1 expression on tumor-specific T cells in blood and tumor were shown. **(b)** Immunofluorescence analysis of PD-1Ab21 distribution in tumors. C57BL/6 mice were transferred with 1×10^6 naïve $CD90.1^+$ OT-1 cells 1 day before inoculation (s.c.) of B16-OVA cells. The mice were immunized with poly I:C and $OVA_{257\text{-}264}$ on day 6, followed by i.p. injections of 100 μg of IL-21Flag or PD-1Ab21 on day 9 after tumor inoculation. Tumors were harvested at 12 hours post protein injection, and stained with anti-CD8 and anti-Flag antibodies. Nuclei were stained with DAPI (blue). Image magnification,×60; scale bar, 50 μm. One representative experiment out of two independent experiments is shown.

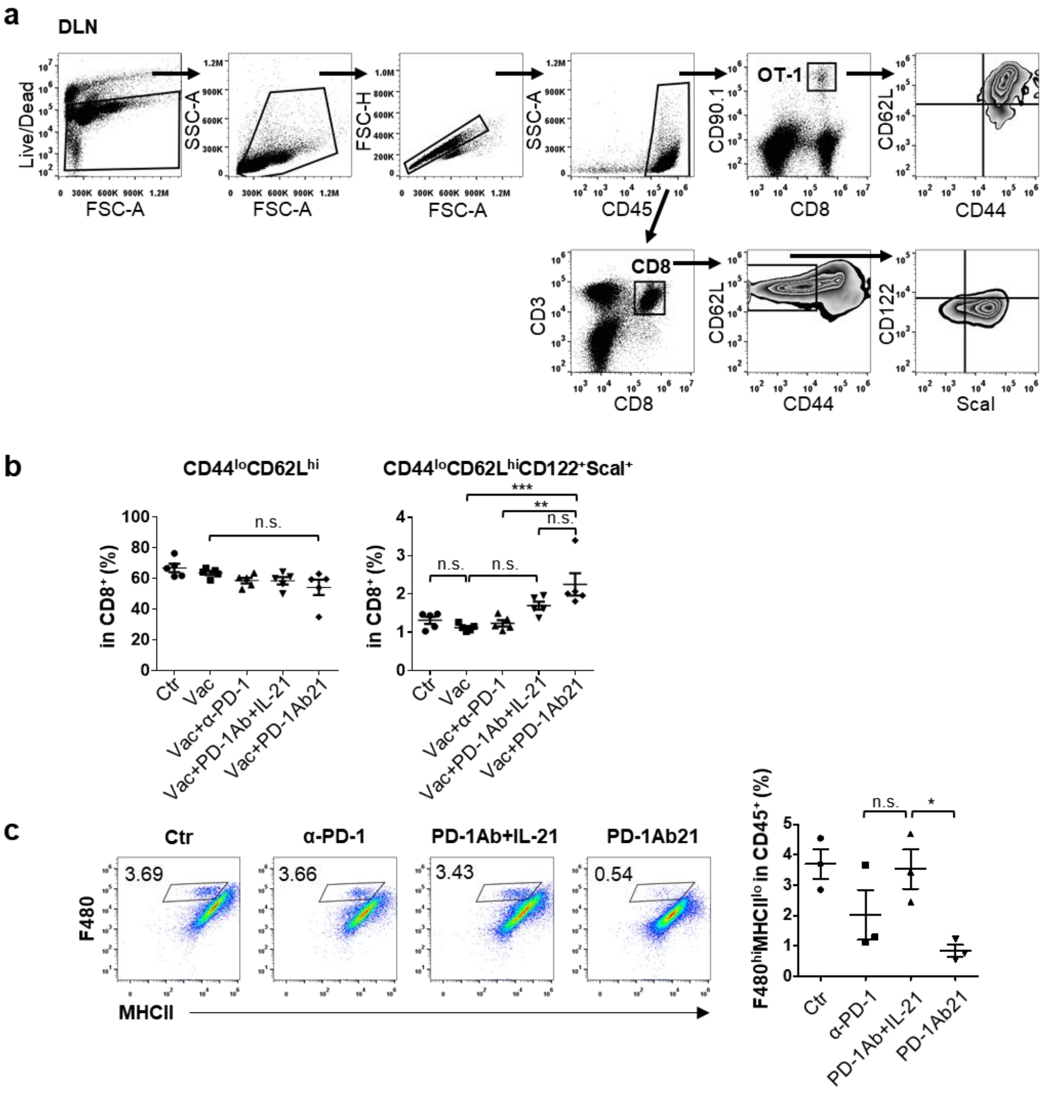

Supplementary Figure 7. Flow cytometry analysis of immune cells in the treated tumor-bearing mice. (a) Gating strategies of $CD8^+$ T cells in DLNs of tumor-bearing mice were shown. Gating strategies of spleen cells were the same as those of DLNs (data not shown). **(b, c)** B16-OVA **(b) or** CT26 **(c)** tumor-bearing mice were treated and tissues were harvested as described in Fig 6. **(b)** Percentages of $CD8^+$ T cells with naïve phenotype and T_{SCM} cells in DLNs (n=5 mice /group). **(c)** Frequencies of macrophages in tumors (n=3 mice /group).

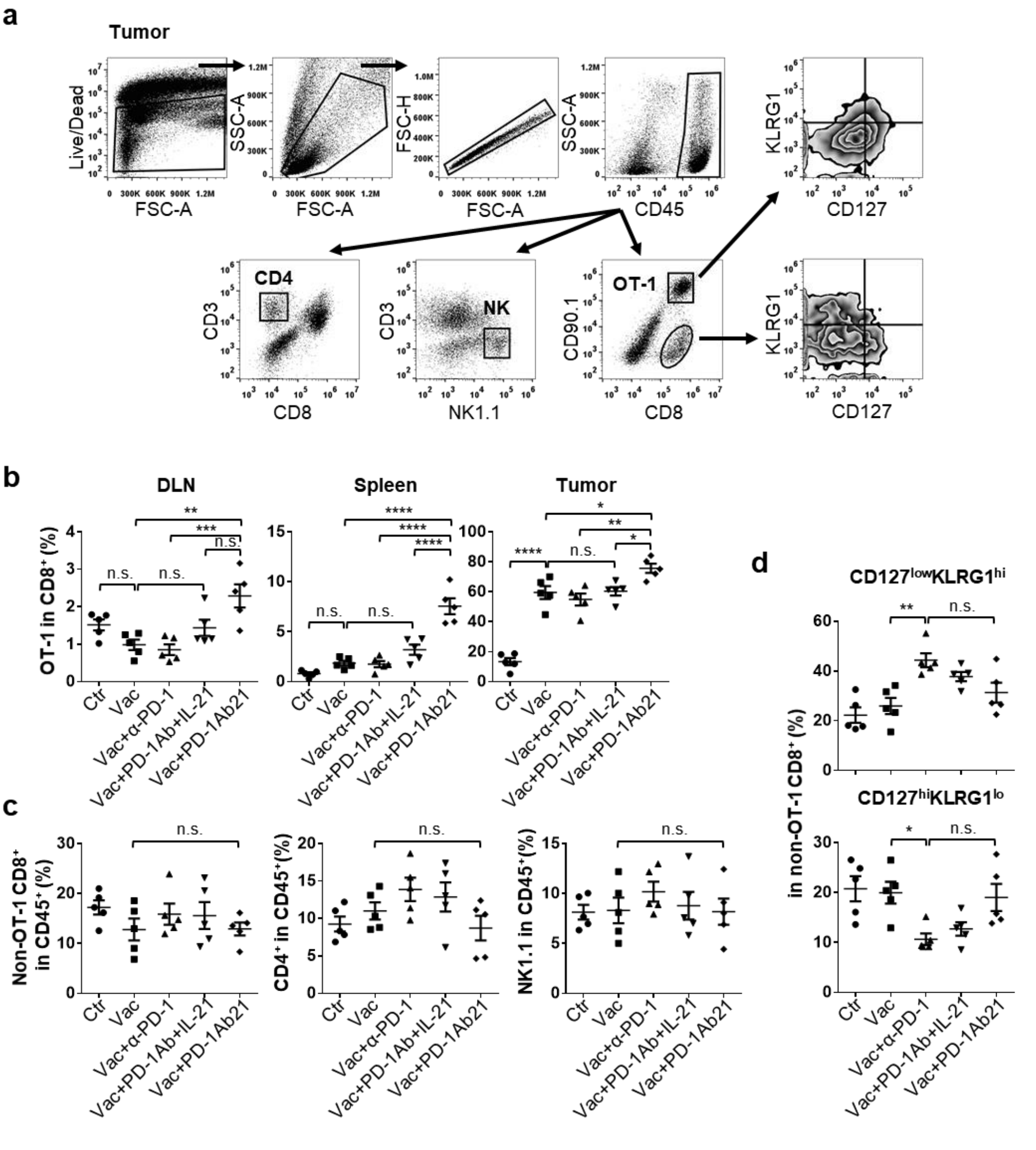

Supplementary Figure 8. Flow cytometry analysis of tumor-specific T cells in the treated tumor-bearing mice. B16-OVA tumor-bearing mice were treated and tissues were harvested as described in Fig 6 (n=5 mice /group). **(a)** Gating strategies of $CD8^+$ T cells in tumor-bearing mice was shown. **(b)** Frequencies of OT-1 cells in DLN, spleen and tumor. **(c)** Frequencies of non-OT-1 $CD8^+$ T cells, $CD4^+$ T cells and NK cells in tumors. **(d)** Phenotypes of non-OT-1 $CD8^+$ T cells in tumors. Data are presented as mean ± SEM and are representative of three independent experiments. Results were compared using one-way ANOVA followed by Tukey's multiple comparison test. (a) **p=0.001, ***p=0.0003. (b) *p=0.0475. (c) ****p<0.0001. DLN: **p=0.0015, ***p=0.0005; Tumor: **p=0.0029, *p=0.0245 (Vac vs. Vac+PD-

1Ab21), *p=0.0352 (Vac+PD-1Ab+IL21 vs. Vac+PD-1Ab21). (e) **p=0.0036, *p=0.0354. n.s., not significant.

Supplementary Table 1

Primer	Sequence
PD1Ab-V_H-F	ACTCGGGTTTAAACGGCCACTTGCCATGAACT
PD1Ab-V_H-R	ACCGCCACTGGACCCTGTTGTTGTGGTTGAGGAGAC
PD1Ab-V_L-F	GGGTCCAGTGGCGGTCAGTTTGTGCTAACTCAGCC
PD1Ab-V_L-R	GCTACTACCGCTTGAGGAAGATGTGACTTTGGGAGAAG ACTT
PD1Ab-V_L-Flag-R	GCAGCGGATCCCTACTTGTCATCGTCGTCCTTGTAATCT GTGACTTTGGGAGAAGACT
PD1Ab- V_L-Linker-R	ACCTTCGCTACCACCGCCCTCGCTGCCGCCGCCTTCGGA GCCGCCACCTTCAGAACCGCCGCCAGAACCACCTGTGA CTTTGGGAGAAGACTT
Linker-IL-21-F	GAGGGCGGTGGTAGCGAAGGTGGTGGCTCCGAGGGTG GCGGTTCCGGCGGCGGTAGCCAAGGGCCAGATCGCCT
IL-21-3xFlag-R	CTTGTAATCGATGTCATGATCTTTATAATCACCGTCATGG TCTTTGTAGTCGGAGAGATGCTGATGAAT
3xFlag-R	GGCAGCAAGCTTCTACTTGTCATCGTCATCCTTGTAATC GATGTCAT
IL-21R-F	CCGGAATTCATGCCCCGGGGCCCAGTGGCT
IL-21R-R	GGAAGATCTGAGCCAGACCCGTCCCAGCCTGCCTCGGG CT
