# Supplementary material for: Targeting IL-21 to tumor-reactive T cells enhances memory T cell responses and anti-PD-1 antibody therapy
Source: Nat Commun. 2021 Feb 11;12:951. doi: 10.1038/s41467-021-21241-0 (PMC7878483; doi:10.1038/s41467-021-21241-0)
Supplement: Supplementary file 3 — Reporting Summary [file 41467_2021_21241_MOESM3_ESM.pdf]

nature research

Corresponding author(s): Shendian Wang AND Ying Li

Last updated by author(s): Jan 15, 2021

Reporting Summary

Nature Research wishes to improve the reproducibility of the work that we publish. This form provides structure for consistency and transparency in reporting. For further information on Nature Research policies, see Authors & Referees and the Editorial Policy Checklist .

Statistics

For all statistical analyses, confirm that the following items are present in the figure legend, table legend, main text, or Methods section.

n/a	Confirmed	
☐	☒	The exact sample size (*n*) for each experimental group/condition, given as a discrete number and unit of measurement
☐	☒	A statement on whether measurements were taken from distinct samples or whether the same sample was measured repeatedly
☐	☒	The statistical test(s) used AND whether they are one- or two-sided<br>*Only common tests should be described solely by name; describe more complex techniques in the Methods section.*
☐	☒	A description of all covariates tested
☐	☒	A description of any assumptions or corrections, such as tests of normality and adjustment for multiple comparisons
☐	☒	A full description of the statistical parameters including central tendency (e.g. means) or other basic estimates (e.g. regression coefficient) AND variation (e.g. standard deviation) or associated estimates of uncertainty (e.g. confidence intervals)
☐	☒	For null hypothesis testing, the test statistic (e.g. *F*, *t*, *r*) with confidence intervals, effect sizes, degrees of freedom and *P* value noted<br>*Give P values as exact values whenever suitable.*
☒	☐	For Bayesian analysis, information on the choice of priors and Markov chain Monte Carlo settings
☒	☐	For hierarchical and complex designs, identification of the appropriate level for tests and full reporting of outcomes
☒	☐	Estimates of effect sizes (e.g. Cohen's *d*, Pearson's *r*), indicating how they were calculated

Our web collection on statistics for biologists contains articles on many of the points above.

Software and code

Policy information about availability of computer code

Data collection	CytoFLEX, Beckman Coulter (Flow cytometry)<br>STORM confocal microscope (Microscopy)<br>RNA-Seq data was collected using BGISEQ-500(BGI,Shenzhen,China)
Data analysis	For the gene expression analysis, the matched reads were calculated and then normalized to RPKM using RESM software (Bo Li et al, BMC Bioinformatics, 2011); Hierarchical clustering analysis and heatmap visualization was performed with Morpheus (https://software.broadinstitute.org/morpheus); Principal component analysis (PCA) was performed using the built-in R function prcomp() (R software v3.5.1); Differential expression analysis was performed using Bioconductor package DESeq2 available at https://github.com/mikelove/DESeq2. FlowJo X 10.0.7 was used for flow cytometry analysis. GraphPad Prism 7.0, RESM software and Microsoft Office were used for data analysis.

For manuscripts utilizing custom algorithms or software that are central to the research but not yet described in published literature, software must be made available to editors/reviewers. We strongly encourage code deposition in a community repository (e.g. GitHub). See the Nature Research guidelines for submitting code & software for further information.

Data

Policy information about availability of data

All manuscripts must include a data availability statement. This statement should provide the following information, where applicable:

- Accession codes, unique identifiers, or web links for publicly available datasets
- A list of figures that have associated raw data
- A description of any restrictions on data availability

The data generated and analyzed during the current study are available from the corresponding author upon reasonable request.

Field-specific reporting

Please select the one below that is the best fit for your research. If you are not sure, read the appropriate sections before making your selection.

- [x] Life sciences
- [ ] Behavioural & social sciences
- [ ] Ecological, evolutionary & environmental sciences

For a reference copy of the document with all sections, see nature.com/documents/nr-reporting-summary-flat.pdf

Life sciences study design

All studies must disclose on these points even when the disclosure is negative.

Sample size	No statistical methods were used to predetermine sample size. For animal experiments at least 5 mice were used per group. All cell culture experiments were performed at least 3 times.
Data exclusions	No samples, mice or data points were excluded from the reported analyses.
Replication	Each experiment including animal and cell culture was repeated at least 3 times as described in Figure legends. Immunofluorescent staining was performed for 2 times. Experimental findings were reliably reproduced between these experiments.
Randomization	Mice used in this experiment were randomly assigned to different groups.
Blinding	Investigators were not blinded, since the administrations and sample collection and processing carried out by the same researchers.

Reporting for specific materials, systems and methods

We require information from authors about some types of materials, experimental systems and methods used in many studies. Here, indicate whether each material, system or method listed is relevant to your study. If you are not sure if a list item applies to your research, read the appropriate section before selecting a response.

Materials & experimental systems

n/a	Involved in the study	
☐	☒	Antibodies
☐	☒	Eukaryotic cell lines
☒	☐	Palaeontology
☐	☒	Animals and other organisms
☒	☐	Human research participants
☒	☐	Clinical data

Methods

n/a	Involved in the study	
☒	☐	ChIP-seq
☐	☒	Flow cytometry
☒	☐	MRI-based neuroimaging

Antibodies

Antibodies used	The following antibodies were used for the flow cytometry analysis: phycoerythrin (PE)- and Cy7-conjugated anti-CD8 (clone 53-6.7; eBioscience), allophycocyanin (APC)-conjugated anti-CD3 145-2C11; BioLegend), fluorescein isothiocyanate (FITC)-conjugated-CD25(clone PC 61; eBioscience), APC-anti-CD44 (clone IM7; eBioscience), PE-anti-CD62L (clone M1L-14; Tonbo), PE-anti-CD122 (clone TM-b1; eBioscience), PE-anti-CD127 (clone A7R34; BioLegend), PerCP- and Cy5.5-conjugated-Scal (clone D7; eBioscience), APC-anti-KLRG1 (clone 2F1; BioLegend), PE-anti-NK1.1(clone PK136, eBioscience), FITC-anti-PD-1(clone J43; eBioscience), PE-anti-B7H1(clone 10F.9G2), PerCP-Cy5.5-anti-CD45(clone 30-F11; BioLegend), FITC-anti-CD90.1(clone HIS51; eBioscience), APC-anti-Flag(clone L5; BioLegend), PE-Cy7-anti-Bcl2 (clone 10C4; eBioscience), PE-anti-TCF1(clone 14456S; Cell Signaling Technology), FITC-anti-CD95 (clone SA367H8, BioLegend), PE-anti-IFN-γ (clone XMG1.2; eBioscience), APC-anti-IL-2 (clone JES6-5H4; eBioscience).<br>For Immunofluorescence staining, CD8α(Santa Cruz Biotechnology), anti-Flag (Sigma) were used.
Validation	All commercially available antibodies are validated by the manufactures.

Eukaryotic cell lines

Policy information about cell lines

Cell line source(s)	TUBO cell line and 7.16.4 hybridoma cells were provided by Yang-Xin Fu (University of Texas Southwestern Medical Center, Dallas, TX). CT26 cell line, B16-OVA cell line, MC38 cell line and G4 hybridoma cells were provided by Dr. Lieping Chen (Yale University School of Medicine, New Haven, CT). Baf3 cell line was bought from BNCC, China.

Authentication	Hybridoma cells were regularly authenticated by checking the production and function of the antibodies; Tumor cell lines were authenticated by morphology, proliferation in vitro and tumorigenicity in vivo.
Mycoplasma contamination	All cell lines were tested negative for mycoplasma.
Commonly misidentified lines (See ICLAC register)	None were used.

Animals and other organisms

Policy information about studies involving animals; ARRIVE guidelines recommended for reporting animal research

Laboratory animals	The following strains of mice were used: C57BL/6 mice ; Balb/c mice; OT-1 transgenic mice; OT-1/Thy1.1 mice; PD-1 KO mice. 6-8-weeks-old female mice were used in the experiments.
Wild animals	The study did not involve wild animals.
Field-collected samples	The study did not involve field-collected samples.
Ethics oversight	All experiments were approved by the Institutional Animal Care and Use Committee of the Institute of Biophysics, CAS.

Note that full information on the approval of the study protocol must also be provided in the manuscript.

Flow Cytometry

Plots

Confirm that:

- [x] The axis labels state the marker and fluorochrome used (e.g. CD4-FITC).
- [x] The axis scales are clearly visible. Include numbers along axes only for bottom left plot of group (a 'group' is an analysis of identical markers).
- [x] All plots are contour plots with outliers or pseudocolor plots.
- [x] A numerical value for number of cells or percentage (with statistics) is provided.

Methodology

Sample preparation	Spleens, draining lymph nodes or tumors were mechanically dissociated. Tumor suspensions were treated with DNAse and collagenase.
Instrument	CytoFLEX, Beckman Coulter
Software	Collection: CytExpert software 2.3<br>Analysis: FlowJo X 10.0.7
Cell population abundance	Purity of post-sort samples were 95% or higher as determined by flow cytometry.
Gating strategy	Cells were first gated on live cells using a viability dye, followed by gating intact cells using FSC/SCC, doublets were excluded using FSC-H vs FSC-A. Cells were then analyzed by cell type specific gating using fluorescently-labeled antibodies.

- [x] Tick this box to confirm that a figure exemplifying the gating strategy is provided in the Supplementary Information.
